# Investigation of the effect of quercetin on experimental traumatic cardiac injury in rats

**DOI:** 10.3389/fcvm.2025.1683944

**Published:** 2025-11-05

**Authors:** Muhammed Enes Taysi, Mustafa Enes Demirel, Ayhan Cetinkaya, Aslihan Saylan, Seyit Ali Kayis, Murat Alisik

**Affiliations:** ^1^ Division of Emergency Medicine, Cankiri State Hospital, Cankiri, Türkiye; ^2^ Department of Emergency Medicine, Faculty of Medicine, Bolu Abant Izzet Baysal University, Bolu, Türkiye; ^3^ Department of Interdisciplinary Biotechnology, Institute of Graduate Education, Bolu Abant Izzet Baysal University, Bolu, Türkiye; ^4^ Department of Physiology, Faculty of Medicine, Bolu Abant Izzet Baysal University, Bolu, Türkiye; ^5^ Department of Histology and Embryology, Faculty of Medicine, Bolu Abant Izzet Baysal University, Bolu, Türkiye; ^6^ Department of Biostatistics and Medical Informatics, Faculty of Medicine, Bolu Abant Izzet Baysal University, Bolu, Türkiye; ^7^ Department of Medical Biochemistry, Faculty of Medicine, Bolu Abant Izzet Baysal University, Bolu, Türkiye

**Keywords:** antioxidant, blunt cardiac injury, interleukin-33, malondialdehyde, quercetin, rat model

## Abstract

**Background:**

Cardioprotection is an important aspect of preventive medicine. Quercetin, a plant-derived flavonoid with antioxidant and anti-inflammatory properties, has been linked to reduced cardiovascular risk.

**Objective:**

To investigate the cardioprotective effects of quercetin in rats with traumatic cardiac injury (TCI).

**Methods:**

Fifty-two Wistar Albino rats were randomly divided into six groups: control (*n* = 7); TCI only (*n* = 9); TCI + DMSO (*n* = 6); and TCI + quercetin at 10, 20, or 40 mg/kg (*n* = 9 each). Quercetin or DMSO was given intraperitoneally at 0.5, 12, and 24 h after trauma. Cardiac trauma was induced by dropping a standardized weight on the chest. Serum biochemical parameters (GPx, SOD, IL-1, IL-33, sST2, MDA) were measured by ELISA, and histopathological damage was scored semiquantitatively. Data were analyzed using ANOVA or Kruskal–Wallis tests with *p* < 0.05 as significant.

**Results:**

GPx elevation was detected only at 10 and 20 mg/kg (vs. TCI; *p* < 0.05); 40 mg/kg was non-significant (*p* > 0.05). Overall, IL-1 differed among groups (*p* = 0.008), with no pairwise comparisons significant after correction (all *p* > 0.05). For IL-33, an overall group effect was observed (*p* = 0.025), while adjusted pairwise tests did not show a consistent between-group pattern (*p* > 0.05). In contrast, malondialdehyde (MDA) levels were significantly reduced, particularly at the highest dose of 40 mg/kg (*p* < 0.05). Superoxide dismutase (SOD) and soluble suppression of tumorigenicity-2 (sST2) levels showed no significant differences among groups (*p* > 0.05). Histopathological evaluation demonstrated that quercetin mitigated myocardial degeneration, inflammatory infiltration, edema, vascular congestion, hemorrhage, and necrosis in a dose-dependent manner, with the most pronounced protective effects observed at 40 mg/kg (*p* < 0.05).

**Conclusions:**

Quercetin, especially at 40 mg/kg, may help prevent secondary cardiac injury after trauma by reducing oxidative stress and limiting histopathological damage. These results support quercetin's cardioprotective potential and warrant confirmation in larger preclinical models with broader designs.

## Introduction

Traumatic cardiac injury (TCI) is among the traumatic diseases that can spontaneously regress or have a potentially fatal course. It is estimated that the cardiac tissue, which is vulnerable to trauma due to its anatomical location, has a high incidence of fatal injury. It has been reported that more than 90% of patients die before reaching the hospital and the survival rate of patients who reach the hospital is between 20% and 75%. This variability has been attributed to challenges in early recognition and delays in accurate diagnosis and management reported across studies. Blunt cardiac injuries (BCI), which can be blunt or penetrating, are caused by motor vehicle accidents (50%), pedestrians struck by motor vehicles (35%), motorcycle accidents (9%) and falls from significant heights (6%) (1, 2).

Traumatic cardiac injury, which may lead to myocardial contusion, hematoma, ventricular rupture, ventricular septal defect, and valve damage or reduced coronary blood flow associated with vascular lesions, subintimal hemorrhage, intraluminal thrombosis, or vasoconstriction, may cause potentially severe hemodynamic compromise and life-threatening arrhythmias. Correct management is crucial; however, the current treatment of cardiac contusion remains mainly supportive, involving fluid replacement, inotropic agents, and antiarrhythmic drugs. To date, no specific or targeted treatment approach has been established for myocardial injury (3, 4).

Oxidative stress plays a central role in the pathophysiology of traumatic cardiac injury. Mechanical trauma leads to excessive generation of reactive oxygen species (ROS) and suppression of endogenous antioxidant systems such as superoxide dismutase (SOD), catalase, and glutathione peroxidase (GPx), resulting in lipid peroxidation, mitochondrial dysfunction, and secondary myocardial damage. Similar oxidative mechanisms have been demonstrated in ischemia–reperfusion injury, where increased ROS production and decreased antioxidant capacity contribute to cardiac dysfunction (5).

The interleukin-33 (IL-33)/suppression of tumorigenesis 2 (ST2) axis has been reported to have anti-inflammatory and anti-proliferative effects in many diseases. It promotes wound healing and tissue repair in immunity and cell homeostasis. IL-33/ST2 is a biomechanical system stimulated during cardiac stretch. When released into the extracellular space by activated cardiac fibroblasts and cardiomyocytes during cardiac stretch responses and by binding the transmembrane isoform of ST2, it exerts cardioprotective effects in both physiological and pathological conditions (6).

sST2 secreted from myocardial tissue under stress has been negatively associated with a worse disease phenotype including adverse remodeling, fibrosis, cardiac dysfunction and impaired hemodynamics and higher risk of progression (6). IL-33, together with ST2, protects against hypertrophy and fibrosis by acting on cardiac myocytes and is mediated by a ligand-receptor mechanism (7, 8). Recent evidence further suggests that sST2 is not only a prognostic biomarker but may also function as a pathogenic mediator of adverse remodeling and fibrosis, as shown in myocardial infarction, heart failure, and aortic stenosis (9). Despite the accumulating evidence supporting its pathophysiological significance, the contribution of the IL-33/ST2 axis to traumatic cardiac injury has yet to be systematically investigated, as current research predominantly addresses non-traumatic cardiovascular disorders rather than trauma. Addressing this gap is essential to determine whether IL-33/ST2 activation mediates trauma-related myocardial damage.

Quercetin, a naturally occurring flavonoid, is widely recognized as one of the most potent dietary antioxidants and possesses a broad pharmacological spectrum, encompassing anti-inflammatory, immunomodulatory, anti-fibrotic, antiviral, and cardioprotective activities (10). Within the cardiovascular domain, quercetin has been demonstrated to exert protective effects in diverse pathological contexts, including myocardial infarction, ischemia–reperfusion injury, hypertension, heart failure, and maladaptive cardiac fibrosis, where it mitigates oxidative stress, enhances endothelial function, and preserves myocardial structural integrity. At the mechanistic level, quercetin modulates critical intracellular signaling cascades, most notably NF-κB and Nrf2/ARE, thereby mediating potent antioxidant and anti-inflammatory responses that collectively contribute to its cardioprotective profile (11, 12). These properties, substantiated by both experimental data and ethnopharmacological evidence, underscore its potential clinical relevance in cardiovascular medicine. Nevertheless, the precise molecular mechanisms underlying quercetin's cardioprotective actions remain insufficiently defined. Importantly, while extensive investigations have addressed its effects in non-traumatic cardiovascular disorders such as myocardial infarction and heart failure, its role in traumatic cardiac injury has received little attention. Despite the limited understanding of the potential interplay between quercetin and the IL-33/ST2 axis—a signaling pathway implicated in inflammation, fibrosis, and adverse cardiac remodeling—modulation of this cascade may represent one of the mechanisms underlying quercetin's well-established antioxidant and anti-inflammatory effects, making it an important mechanistic framework for the present study (7, 10). Accordingly, the present study was designed to address this knowledge gap by evaluating the effects of quercetin on the oxidant/antioxidant system, IL-33/ST2 signaling, and histopathological alterations in myocardial tissue following trauma.

## Material and methods

### Experimental design

The study was conducted in accordance with the ARRIVE guidelines (13). Ethical approval was obtained from the Bolu Abant Izzet Baysal University Animal Research Local Ethics Committee (Approval number: 2022/13). Animal experiments were conducted in accordance with the relevant guidelines, study procedures and ethics committees of the animal experiments regulation determined by the Ministry of Agriculture and Forestry.

In the study, 52 male Wistar Albino rats (12–13 weeks of age; weighing 250–300 g) were obtained from Bolu Abant Izzet Baysal University Experimental Animals Application and Research Center and maintained under appropriate conditions during the study. Rats were housed in rooms with a temperature of 17–21 °C and 50%–55% humidity, 12 h of light and 12 h of darkness. They were kept in cages with free access to food and water.

The rats were randomly divided into six groups: the control group included 7 rats, while 9 rats were allocated to each of the remaining groups. However, 3 rats died after trauma in the traumatic cardiac injury (TCI) + dimethyl sulfoxide (DMSO) group and the study was completed with 6 rats in this group. The animals were randomly assigned to the experimental groups using a simple random allocation method prior to trauma induction. Group allocation was performed by an investigator who was not involved in subsequent experimental procedures to ensure allocation concealment. All biochemical and histopathological evaluations were conducted by researchers blinded to the group assignments. The personnel responsible for animal care and the investigators performing sample collection were different from those conducting data analysis, ensuring adherence to the ARRIVE guidelines regarding randomization and blinding.

#### Group I (control)

The first group was classified as the control group and no treatment or medication was applied.

#### Group II (TCI)

In the second group, only TCI model was created and sacrificed on the 3rd day (72nd hour).

#### Group III (TCI + DMSO)

In the third group, TCI model was established and only 0.1 mL DMSO solution was injected intraperitoneally at 0.5, 12 and 24 h.

#### Group IV (TCI + Q10)

In the fourth group, TCI was induced and quercetin; 10 mg/kg was injected intraperitoneally at 0.5, 12 and 24 h after trauma, respectively.

#### Group V (TCI + Q20)

In the fifth group, TCI was induced and quercetin was injected at a dose of 20 mg/kg intraperitoneally at 0.5, 12 and 24 h after trauma, respectively.

#### Group VI (TCI + Q40)

In the sixth group, TCI was induced and quercetin was injected at a dose of 40 mg/kg intraperitoneally at 0.5, 12 and 24 h after trauma.

Based on the *a priori* power analysis, the target sample size was *n* = 9 per group (*n* = 7 for the control group in accordance with ethical approval). Three early deaths occurred in the TCI + DMSO group; in accordance with the ethical approval, no replacement was performed, and analyses for this group were conducted with *n* = 6.

The chronological flow of the experimental protocol is summarized in Table 1.

**Table 1:** Experimental timeline and intervention scheme for all study groups.

Groups/Hours	0 h	0.5 h	12 h	24 h	72 h
Control	–	–	–	–	Sampling
TCI	Trauma	–	–	–	Sampling
TCI + DMSO	Trauma	↓	↓	↓	Sampling
TCI + Q10	Trauma	↑	↑	↑	Sampling
TCI + Q20	Trauma	↑↑	↑↑	↑↑	Sampling
TCI + Q40	Trauma	↑↑↑↑	↑↑↑↑	↑↑↑↑	Sampling

Traumatic cardiac injury (TCI) was induced at 0 h in all animals except the control group. Treatment injections were administered intraperitoneally at 0.5 h, 12 h, and 24 h as follows.

• After trauma induction, no pharmacological intervention was performed in the Control (Group 1) or TCI group (Group 2).

• ↓ (DMSO) – vehicle control, a single dose at each time point (Group 3).

• ↑ (Q10) – quercetin 10 mg/kg, (Group 4).

• ↑↑ (Q20) – quercetin 20 mg/kg, (Group 5).

• ↑↑↑↑ (Q40) – quercetin 40 mg/kg, (Group 6).

Whole blood and myocardial tissue sampling was performed 72 h later in all groups.

DMSO, dimethyl sulfoxide; Q, quercetin; h, hour.

In this study, quercetin (purity ≥98%, Sigma-Aldrich, USA) was dissolved in dimethyl sulfoxide (DMSO) at a concentration of 1%. The solution was freshly prepared before each injection and administered intraperitoneally without further dilution (14).

In designing our study, we referred to prior experimental work in which quercetin was administered at different doses and with varying timing protocols in diverse injury models (15, 16). While these investigations provided guidance regarding dose selection and administration schedules, they were limited either to other organ systems or to a single fixed dose approach. In contrast, our study applied a post-trauma administration strategy with graded doses (10, 20, and 40 mg/kg), integrating insights from previous literature into a novel experimental design. This allowed us to explore both the dose–response relationship and the therapeutic potential of quercetin in a trauma-induced cardiac injury model, an area where data have been lacking.

**Figure 1:**
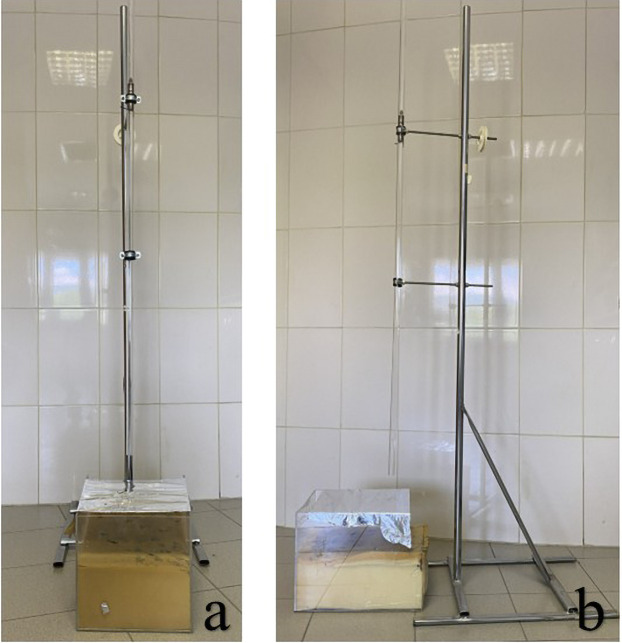
**(a)** Front view and **(b)** side view of the trauma model used to apply controlled cardiac trauma.

**Figure 2:**
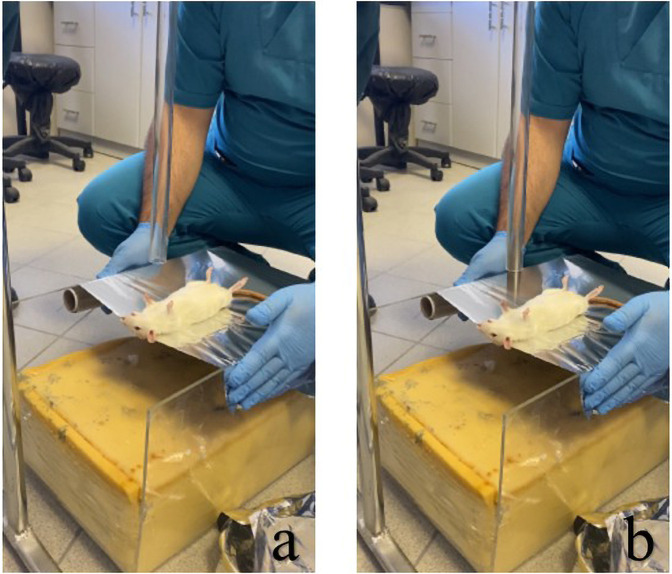
Modeling of the rat for induction of cardiac trauma and subsequent application of trauma: **(a)** positioning of the rat for induction of injury, and **(b)** process of dropping the weight on the rat.

The solution was freshly prepared prior to each intraperitoneal injection and administered immediately without dilution. The trauma model developed by Raghavendran et al. was employed (17). In this modeling, cardiac trauma was induced by dropping a hollow aluminum cylindrical weight (0.25 kg) through a vertical stainless tube on a Lexon platform from a height of 1 meter onto the rib cage (Figures 1, 2). Prior to impact, the area of the platform where the weight would fall was stabilized and supported with aluminum foil to create a 5 cm clearance to prevent recoil, minimize friction, and optimize energy transfer to the anesthetized animal (17).

Seventy-two hours after trauma induction, anesthesia was induced by using ketamine (90 mg/kg) and xylazine (10 mg/kg) intramuscularly. Following anesthesia, the rats were placed in the prone position and stabilized. After shaving the abdomen and thorax of the rats and cleaning the area with 10% povidone-iodine, the skin was dissected with a linear incision over the sternum, following the median line, starting just distal to the xiphoid. After the diaphragm was removed, the heart tissue was observed and 5 mL of intracardiac blood was taken from the right ventricle and the animals were sacrificed. The heart tissue was then excised for further analysis. For histopathological analysis, myocardial tissue samples were obtained from the ventricular region.

### Pre-specified biological domains and endpoints

The study was designed to investigate two predefined biological domains: (i) oxidative stress, and (ii) the IL-33/sST2 axis. Within the oxidative-stress domain, the primary outcome variable was serum glutathione peroxidase (GPx) at 72 h, with malondialdehyde (MDA) serving as a key secondary marker in the same domain. The IL-33/sST2 axis was evaluated as an exploratory mechanistic objective, as a powered difference in sST2 levels was not anticipated *a priori* given its role as a decoy receptor and prior evidence that, under antioxidant modulation, sST2 expression typically remains unchanged or is downregulated.

### Sample size and power analysis

An *a priori* sample-size calculation was performed before study initiation (G*Power v3.1). Pilot control data indicated GPx 17 ± 4. Assuming a 40% increase with quercetin (Cohen's d ≈ 1.70), *n* = 9 per group was required to achieve *α* = 0.05 (two-tailed) and 1–*β* = 0.95. Accordingly, *n* = 9 per group was the target sample size for all trauma-exposed and treatment groups.

### Biochemical analysis

Blood was collected in tubes containing no anticoagulant and allowed to clot for 30 min at room temperature. Serum samples were then centrifuged at 1,500×g for 10 min at 4 °C and stored at −80 °C until analysis. GPx, SOD activities, MDA, IL-1, IL-33 and sST2 levels in serum samples of rats were determined by ELISA method using a commercial kit (BT LAB, 501 Changsheng S Ed, Nanhu Dist, Jiaxing, Zhejiang, China) and the color intensity was read at 450 nm with an ELISA reader (Biotek ELx800).

### Histopathological analysis

Heart tissues were rapidly dissected from the euthanized rats and fixed with 10% neutral buffered formalin solution for 48 h for histological examination. Tissue samples were dehydrated and embedded by adopting the standard protocols. After routine tissue treatment, 3 μm thickness sections were obtained from the paraffin blocks and stained with Hematoxylin and Eosin (Hematoxylin: Beslab, lot: 01072016001- Eosin: Atomscientific, lot: RRSP37/E). The sections were observed under light microscope (Nikon eclipse 80i) for histological changes (inflammatory cell infiltration, vascular congestion, intramyocardial hemorrhage, necrosis, myocardial edema, myocardial degeneration) and the photographs were taken by digital camera. The histopathological damage severity in cardiac tissues section were scored semiquantitatively between 0 and 3. No change was scored as 0, mild damage was scored as 1, moderate damage was scored as 2, and severe damage was scored as 3 (18). Each section was evaluated by a single histologist in a blinded manner, with group assignments concealed during the assessment (coded evaluation).

### Statistical analysis

Statistical analysis of the data was performed by using the R software (19). The Shapiro–Wilk test was used to check the conformity of continuous variables to normal distribution. Continuous variables, showing normal distribution, were expressed mean ± SD for descriptive statistics while non-normally distributed continues variables expressed as median (min.-max.). One-way Analysis of Variance (ANOVA) was used for group comparison, for normally distributed variables, and Tukey test was used as *post-hoc* test. For non-normally distributed variables, Kruskal–Wallis test was used for multiple comparisons, Mann Whitney U test was employed as *post-hoc* test and Bonferroni correction was made. *P* values <0.05 were considered statistically significant.

## Results

### Biochemical parameters

All measured biochemical parameters are presented in Table 2. Serum GPx activities of the TCI group were found to be statistically significantly lower when compared with the TCI + Q10 and TCI + Q20 groups (*p* < 0.05). In addition, no statistically significant difference was found between TCI and control, TCI + DMSO and TCI + Q40 groups (*p* > 0.05). Regarding SOD activities, no statistically significant differences were detected among the groups (*p* > 0.05).

**Table 2:** Statistical results of measured biochemical parameters.

Group/Biochemical parameters	Control	TCI	TCI + DMSO	TCI + Q10	TCI + Q20	TCI + Q40	*P* value
	*n*:7	*n*:9	*n*:6	*n*:9	*n*:9	*n*:9	
GPx (ng/mL)	17.2 ± 3.5^b^	15.7 ± 1.3^b^	19.2 ± 5.9^ab^	24.3 ± 4.3^a^	23.8 ± 6.0^a^	20.9 ± 1.9^ab^	<0.001^£^
SOD (ng/mL)	2.1 ± 0.3	2.0 ± 0.3	1.9 ± 0.2	1.9 ± 0.6	1.7 ± 0.4	1.9 ± 0.4	0.596^£^
IL-1 (pg/mL)	22.9 (19.8–25.7)	24.6 (22.2–28.1)	24.0 (19.8–27.3)	27.9 (14.3–44.0)	27.8 (17.7–37.6)	29.3 (21.4–32.7)	0.008^££^
IL-33 (ng/L)	48.3 ± 14.6^b^	60.4 ± 15.0^ab^	72.8 ± 8.2^a^	66.6 ± 13.6^ab^	63.8 ± 12.7^ab^	58.6 ± 10.5^ab^	0.025^£^
sST2 (ng/mL)	15.2 (13.6–19.9)	14.8 (12.7–17.1)	15.6 (10.4–17.2)	15.7 (12.6–20.1)	14.8 (11.6–19.5)	15.6 (13.2–23.0)	0.974^££^
MDA (nmol/mL)	1.1 (1.0–1.2)^a^	1.4 (1.2–1.5)^b^	0.9 (0.8–1.1)^a^	1.0 (0.5–1.4)^ab^	1.1 (0.4–1.6)^ab^	1.1 (1.0–1.2)^a^	<0.001^££^

^£^ The *p* value was obtained via one-way analysis of variance. Values are mean ± standard deviation. Tukey test was used for pairwise comparisons. Groups that do not share the same superscript in a row are statistically significantly different from each other (*p* < 0.05).

^££^ The *p* value was obtained by the Kruskal–Wallis test. Values are median value (minimum-maximum). Mann Whitney U test was used for pairwise comparisons and Bonferroni correction was made. Groups that do not share the same superscript in a row are statistically significantly different from each other (*p* < 0.05).

The overall analysis showed a difference in IL-1 levels among groups (*p* = 0.008), but no pairwise comparisons were statistically significant (*p* > 0.05). No significant differences were observed among the groups for IL-33 levels (*p* > 0.05), except for higher IL-33 levels in the TCI + DMSO group compared with the control group (*p* < 0.05). For sST2, no statistically significant intergroup differences were observed (*p* > 0.05).

MDA levels were significantly higher in the TCI group than in the control group (*p* < 0.05). No significant differences were detected between the TCI group and the TCI + Q10 or TCI + Q20 groups (*p* > 0.05). A significant reduction in MDA levels was observed in the TCI + Q40 group compared with the TCI group (*p* < 0.05).

### Histopathologic results

**Figure 3:**
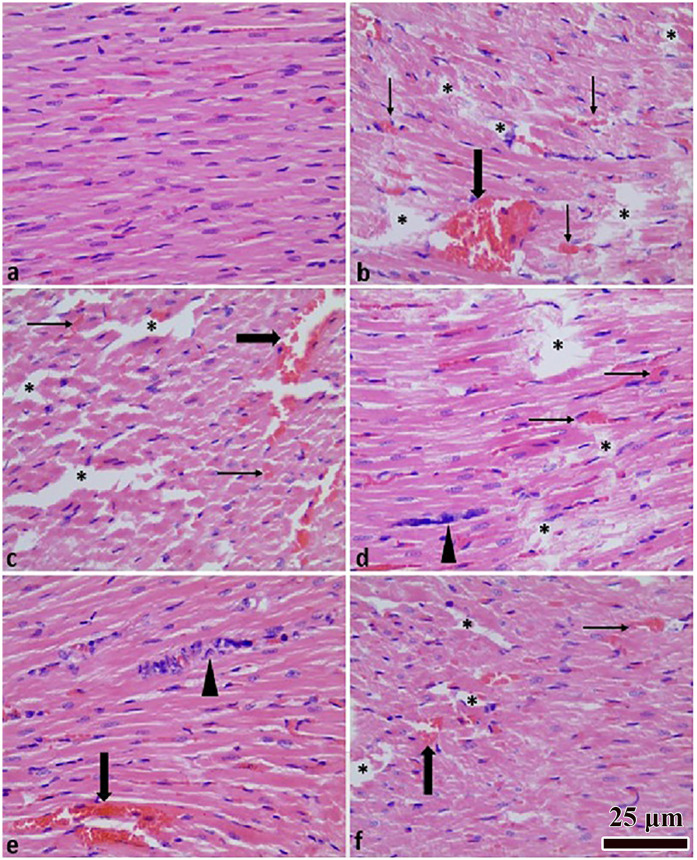
Heart tissue histopathological images. Normal histological appearance **(a)**; TCI group **(b)**; TCI + DMSO group **(c)**; TCI + Q (10 mg) group **(d)**; TCI + Q (20 mg) group **(e)**; TCI + Q (40 mg) group **(f)** Myocardial degeneration (*), inflammation (arrow head), congestion (thick arrow), hemorrhage (thin arrow). H&E staining × 400 magnification.

Histopathological evaluation of intramyocardial hemorrhage, myocardial degeneration, inflammatory cell infiltration, myocardial edema, vascular congestion, and necrosis revealed minimal or absent severity in the control group and marked increases in the trauma group (overall *p* < 0.001; Table 3). Quercetin treatment attenuated these alterations in a dose-dependent manner, showing statistically significant reductions in tissue damage compared with the trauma group (Table 3). Representative microscopic images of myocardial tissue from each group are shown in Figure 3.

**Table 3:** Statistical analysis results of histopathological scoring.

Group/Histopathological scoring	Control	TCI	TCI + DMSO	TCI + Q10	TCI + Q20	TCI + Q40	*P* value
	*n*:7	*n*:9	*n*:6	*n*:9	*n*:9	*n*:9	
IMH	0.0 (0.0–1.0)^a^	3.0 (2.0–3.0)^b^	3.0 (1.0–3.0)^bc^	2.0 (1.0–3.0)^bc^	1.0 (0.0–2.0)^ac^	1.0 (1.0–2.0)^ac^	<0.001^££^
VC	0.0 (0.0–0.0)^a^	3.0 (3.0–3.0)^b^	3.0 (2.0–3.0)^bc^	3.0 (2.0–3.0)^b^	2.0 (0.0–2.0)^c^	1.0 (0.0–2.0)^c^	<0.001^££^
ICI	0.0 ± 0.0^c^	2.3 ± 0.9^a^	2.5 ± 0.8^a^	1.7 ± 0.7^ab^	1.2 ± 0.7^b^	0.8 ± 0.7^bc^	<0.001^£^
ME	0.1 ± 0.4^b^	1.9 ± 0.9^a^	2.0 ± 0.9^a^	1.7 ± 1.0^a^	0.4 ± 0.5^b^	0.4 ± 0.5^b^	0.001^£^
N	0.0 (0.0–0.0)^a^	2.0 (1.0–3.0)^bc^	3.0 (2.0–3.0)^b^	2.0 (0.0–3.0)^bc^	1.0 (0.0–2.0)^bc^	1.0 (0.0–2.0)^ac^	<0.001^££^
MD	0.0 (0.0–1.0)^a^	3.0 (2.0–3.0)^b^	3.0 (2.0–3.0)^bc^	3.0 (0.0–3.0)^bc^	2.0 (1.0–3.0)^c^	2.0 (0.0–3.0)^abc^	<0.001^£^

IMH, intramyocardial hemorrhage; VC, vascular congestion; ICI, inflammatory cell infiltration; ME, myocardial edema; N, necrosis; MD, myocardial degeneration.

^£^ *p* value was obtained as a result of one-way analysis of variance. Values are given as mean ± standard deviation. Tukey test was used for pairwise comparisons. Groups that do not share the same superscript in a row show statistically significant differences from each other (*p* < 0.05).

^££^ *p* value was obtained with Kruskal–Wallis test. Values are given as median value (minimum-maximum). Mann Whitney U test was used for pairwise comparisons, Bonferroni correction was applied. Groups that do not share the same superscript in a row show statistically significant differences from each other (*p* < 0.05).

## Discussion

In this experimental study, the cardioprotective effects of quercetin against mechanical cardiac trauma were demonstrated through both biochemical and histological analyses. The use of increasing doses of quercetin resulted in a significantly beneficial reversal of the inflammatory response and oxidative processes in rats, particularly visible in the histological findings. These findings highlight the potential of quercetin to reduce secondary cardiac damage and may inform future therapeutic approaches in trauma-related myocardial injury. We have shown that the negative effects of trauma can be decreased or at least regarded as a curative factor in the healing process following the use of quercetin, in which numerous cascades are involved (15). In a recent review, Zhang et al. summarized that quercetin exerts multiple beneficial effects in cardiovascular diseases, including antioxidant, anti-inflammatory, endothelial-protective, and myocardial-protective actions, thereby supporting its potential cardioprotective role (11).

Studies have shown that oxidative stress, inflammation, and posttraumatic cardiomyocyte apoptosis contribute significantly to secondary cardiac dysfunction following mechanical trauma, which can occur even in the absence of direct cardiomyocyte damage within the initial 24 h after injury. The disruption of the cooperation between unchanged SOD levels and increased GPx activity may lead to the accumulation of non-detoxified superoxide radicals. In our study, the oxidative damage observed in the TCI group, reflected as significantly elevated serum MDA, may be attributable to these reactive radicals. Lipid peroxidation is a well-established indicator of oxidative stress, and MDA measurement is widely accepted as a marker of lipid peroxidation (20, 21). Previous studies have similarly reported that trauma-induced cardiac oxidative stress is associated with reduced antioxidant enzyme activity and increased lipid peroxidation products (5, 22). In this experiment, quercetin treatment at all tested doses (10, 20, and 40 mg/kg) reduced MDA levels compared with the injury group, with statistical significance achieved only in the 40 mg/kg group. This suggests that the antioxidative effect of quercetin becomes more evident at higher doses. In parallel, quercetin administration enhanced antioxidant defense, as reflected by increased GPx activity. Overall, these findings indicate that quercetin exerts a dose-dependent protective effect against traumatic cardiac injury by lowering lipid peroxidation and enhancing antioxidant capacity.

We hypothesize that the increase in GPx enzyme levels observed in the TCI + DMSO group may be attributed to the antioxidant properties of DMSO (23, 24). Regarding the observed decrease in GPx levels in the 40 mg/kg quercetin group despite initial increases at lower doses, this may be due to an earlier activation of the antioxidant response and more rapid recovery. Quercetin at higher doses may have mitigated oxidative stress earlier, which may have led to a reduced need for continued endogenous antioxidant enzyme activity at the 72-hour mark. This is supported by the fact that the 40 mg/kg group showed the most pronounced histopathological improvement. Similarly, the decrease in GPx levels observed in the TCI group compared to the control group may be attributed to the absence of a stimulatory and reparative antioxidant agent. Additionally, considering the complex and multifactorial nature of trauma-related responses, it is also possible that unknown regulatory mechanisms affected GPx levels in this group. Further studies with extended molecular profiling are needed to clarify these dynamics. Upon examining the MDA levels across groups, it was observed that the administration of antioxidant agents led to a reduction in MDA levels, approaching those measured in the control group. This suggests that cardiac repair may have occurred during this period, supported by antioxidant mechanisms. It is likely that multiple pathways are involved in this process, with changes in GPx levels serving as indicators of these underlying mechanisms. Nevertheless, further studies are required to elucidate these complex and not yet fully understood processes (25, 26).

The IL-33/ST2 axis has recently attracted growing attention for its diverse regulatory functions and therapeutic potential across a wide range of diseases, including cancer, fibrosis, autoimmune disorders, and central nervous system pathologies. Acting as an alarmin released upon tissue damage, IL-33 regulates immune responses and tissue repair, and targeted pharmacological strategies against this pathway are already in preclinical development. While these insights highlight the importance of IL-33/ST2 signaling in disease progression, trauma-related myocardial injury remains poorly characterized in this context (27).

Our findings indicated a modest tendency toward higher IL-33 levels in quercetin-treated groups, although this did not reach statistical significance, while sST2 levels remained unchanged. Although the findings obtained do not provide evidence of definitive pathway activation, they may suggest a modest modulatory effect of quercetin on the IL-33/ST2 axis following traumatic cardiac injury. Taken together with our oxidative stress data—namely, a significant reduction in MDA levels at 40 mg/kg and increased GPx activity—these observations raise the possibility that quercetin's antioxidant actions could indirectly influence IL-33/ST2 signaling. However, this hypothesis requires further investigation. To the best of our knowledge, this is the first study to assess quercetin's effects on IL-33/ST2 signaling in a mechanical trauma model. Given the emerging recognition of this axis as a therapeutic target, our results provide preliminary insights but also emphasize the need for additional studies incorporating time-course analyses, tissue-level measurements, and functional assessments to establish mechanistic links (28, 29).

Zhang et al. reported that dihydromyricetin attenuated CCl₄-induced cardiac dysfunction in mice by downregulating IL-33 and ST2 expression, suggesting that inhibition of the IL-33/ST2 signaling pathway may contribute to its cardioprotective effect (30). In our study, quercetin administration in traumatic cardiac injury was considered to be associated with a possible modulation of the IL-33/ST2 axis. However, although this effect did not reach statistical significance, when the study is evaluated as a whole, it may still be regarded as providing valuable insights into a possible activation of the IL-33/ST2 pathway. In this aspect, our findings suggest that quercetin, as a natural flavonoid, may exert cardioprotective effects at least partly through modulation of the IL-33/ST2 signaling pathway, a possibility that warrants further investigation in larger and more comprehensive studies.

IL-1 levels were numerically higher in the quercetin-treated groups compared with the control group, although the differences were not statistically significant. This pattern is consistent with an ongoing inflammatory response observed in the trauma model. Histopathological evaluation demonstrated that inflammatory cell infiltration decreased progressively with increasing quercetin dose. These findings indicate a potential anti-inflammatory effect of quercetin in traumatic cardiac injury, which warrants confirmation through further studies (28).

In histopathological evaluation, the injury group showed greater severity than the control group with respect to intramyocardial hemorrhage, myocardial degeneration, inflammatory cell infiltration, myocardial edema, vascular congestion, and necrosis. Quercetin administration attenuated the severity of these findings in a dose-dependent manner, with statistically significant protective effects observed predominantly at the highest dose (40 mg/kg) (Table 3). When the histopathological results were evaluated collectively, quercetin demonstrated a protective effect against cardiac injury that appeared to be dose related. Consistently, a previous study reported that quercetin treatment alleviated myocardial cell edema, loss of transverse striations, and inflammatory cell infiltration in heat stroke–related cardiac injury (11).

In experimental models of traumatic cardiac injury, mortality rates can vary significantly depending on the severity of the trauma, the method used, and whether any therapeutic interventions are applied. Some experimental models have reported high mortality rates (31), whereas others have reported no deaths (32), depending on the severity of trauma and the use of cardioprotective agents. In our study, three animals died in the TCI + DMSO group shortly after trauma induction, which was anticipated due to the invasive nature of the procedure. However, no mortality was observed in the quercetin-treated groups, which may Of particular interest, anti-inflammatory, and antioxidant properties that help mitigate lethal outcomes in cardiac tissue subjected to trauma. The relatively low mortality observed in our study is consistent with previous reports and likely reflects both the variability inherent in the trauma model and the beneficial effects of quercetin.

The strength of this study lies in its design as an animal experiment, which minimizes confounding factors such as comorbidities frequently encountered in human populations. Moreover, the use of direct cardiac tissue provided a more accurate assessment of oxidative damage in experimental traumatic cardiac injury. The inclusion of histopathological evaluation represents another strength of the study. Nonetheless, the absence of cardiac biomarkers such as troponin T and creatine kinase (CK-MB) constitutes a limitation. In addition, the study was conducted on a relatively small number of rats, and the parameters were therefore evaluated on a limited sample size. Furthermore, functional cardiac assessments such as electrocardiography or echocardiography were not performed. Another limitation is that although our initial plan considered serial sampling to capture temporal dynamics, for ethical and practical reasons, we restricted analyses to a single endpoint at 72 h to minimize animal mortality and preserve statistical power. As a result, while the multiple dosing schedule was intended to cover both early and intermediate phases after trauma, only the cumulative effects at 72 h could be assessed. Therefore, another limitation of this design is that early and late responses cannot be directly assessed. Finally, early attrition in the TCI + DMSO group (final *n* = 6) may have reduced statistical power for some contrasts and should be considered when interpreting non-significant findings.

**Figure 4:**
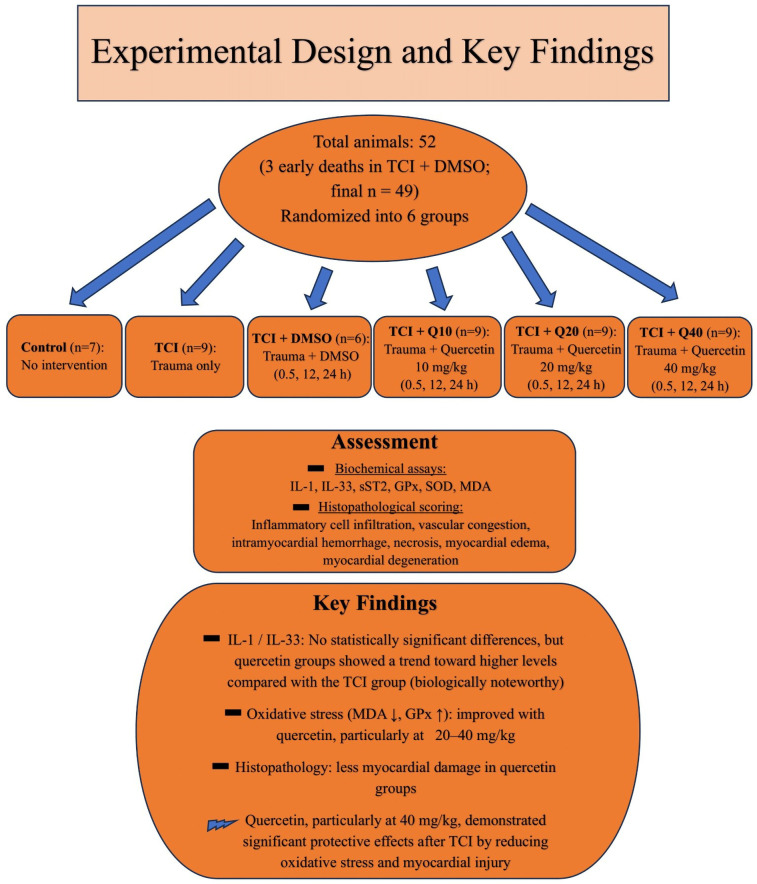
Schematic summary of the study design and key findings.

Figure 4 illustrates the overall study design and summarizes key findings.

## Conclusions

Our study introduces quercetin as a potential therapeutic candidate in traumatic cardiac injury models, primarily through its modulation of oxidative stress and a possible modulatory effect on the IL-33/ST2 pathway, with the most pronounced protective effect observed at the 40 mg/kg dose. These findings suggest a cardioprotective potential of quercetin in the context of blunt chest trauma and may provide a rationale for future translational research on natural compounds in cardioprotection. Nevertheless, given the preclinical nature of this rat model, the findings should be interpreted within the experimental context and warrant confirmation in further studies.

Further research is warranted to: (i) evaluate tissue-level expression of IL-33/ST2 and related signaling mediators; (ii) perform larger-scale studies to confirm the reproducibility and robustness of our findings; (iii) investigate additional biochemical markers of oxidative stress and antioxidant defense beyond MDA, SOD, and GPx, to obtain a more comprehensive view of redox regulation; and (iv) apply advanced histopathological and molecular techniques (e.g., immunohistochemistry, Western blotting, transcriptomic profiling) to elucidate the mechanistic underpinnings. Moreover, time-course experiments and functional cardiac assessments (such as echocardiography) would help to clarify the temporal dynamics and physiological relevance of quercetin's effects.

Collectively, these future directions could advance understanding of quercetin's therapeutic potential and its mechanistic role in trauma-induced myocardial injury.

## Data Availability

The raw data supporting the conclusions of this article will be made available by the authors, without undue reservation.
